# Assessing direct paths of intracortical causal information flow of oscillatory activity with the isolated effective coherence (iCoh)

**DOI:** 10.3389/fnhum.2014.00448

**Published:** 2014-06-20

**Authors:** Roberto D. Pascual-Marqui, Rolando J. Biscay, Jorge Bosch-Bayard, Dietrich Lehmann, Kieko Kochi, Toshihiko Kinoshita, Naoto Yamada, Norihiro Sadato

**Affiliations:** ^1^The KEY Institute for Brain-Mind Research, University of ZurichZurich, Switzerland; ^2^Department of Neuropsychiatry, Kansai Medical UniversityOsaka, Japan; ^3^CIMFAV, Universidad de ValparaisoValparaiso, Chile; ^4^Neuroinformatic Department, Cuban Neuroscience CenterHavana, Cuba; ^5^Department of Psychiatry, Shiga University of Medical ScienceShiga, Japan; ^6^Division of Cerebral Integration, National Institute for Physiological SciencesOkazaki, Japan

**Keywords:** causal intracortical connectivity, LORETA, isolated effective coherence, resting state electriphysiological connectivity, alpha oscillation connectivity

## Abstract

Functional connectivity is of central importance in understanding brain function. For this purpose, multiple time series of electric cortical activity can be used for assessing the properties of a network: the strength, directionality, and spectral characteristics (i.e., which oscillations are preferentially transmitted) of the connections. The partial directed coherence (PDC) of Baccala and Sameshima (2001) is a widely used method for this problem. The three aims of this study are: (1) To show that the PDC can misrepresent the frequency response under plausible realistic conditions, thus defeating the main purpose for which the measure was developed; (2) To provide a solution to this problem, namely the “isolated effective coherence” (iCoh), which consists of estimating the partial coherence under a multivariate autoregressive model, followed by setting all irrelevant associations to zero, other than the particular directional association of interest; and (3) To show that adequate iCoh estimators can be obtained from non-invasively computed cortical signals based on exact low resolution electromagnetic tomography (eLORETA) applied to scalp EEG recordings. To illustrate the severity of the problem with the PDC, and the solution achieved by the iCoh, three examples are given, based on: (1) Simulated time series with known dynamics; (2) Simulated cortical sources with known dynamics, used for generating EEG recordings, which are then used for estimating (with eLORETA) the source signals for the final connectivity assessment; and (3) EEG recordings in rats. Lastly, real human recordings are analyzed, where the iCoh between six cortical regions of interest are calculated and compared under eyes open and closed conditions, using 61-channel EEG recordings from 109 subjects. During eyes closed, the posterior cingulate sends alpha activity to all other regions. During eyes open, the anterior cingulate sends theta-alpha activity to other frontal regions.

## Introduction

The type of problem that we are interested in can best be understood with an informal hypothetical example.

**Figure 1:**
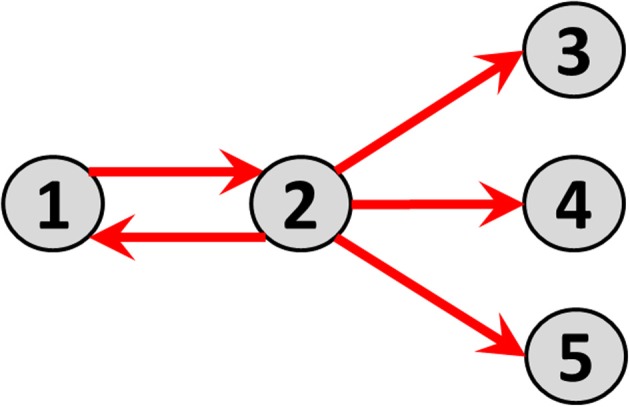
**Direct causal directed connections between nodes, corresponding to the toy example defined by the multivariate autoregressive model in Equation 13**.

Consider time series of local electric potential differences measured at five sites (i.e., nodes) on the cortex (electrocorticogram, ECoG). Before connecting the five nodes, each one in isolation has its distinct activity. For instance, node 1 oscillates at 28 Hz, node 2 at 16 Hz, and nodes 3, 4, and 5 at 23 Hz. In the next construction step, some causal direct and directional connections with measurable time lags are established: node 1 sends to node 2; and node 2 sends identically to nodes 3, 4, and 5. The resulting connectivity graph is shown in Figure 1.

Instantaneous connections, as considered, e.g., by Faes et al. (2013), are not considered in this hypothetical example, i.e., ephaptic conduction is assumed to be absent, see e.g., Weiss et al. (2013).

Note the distinction between “direct” and “indirect” connection paths. Examples in this hypothetical network are: (A) The direct connection path from node 1 to node 2; (B) The indirect connection path from node 1 to node 3 mediated by node 2.

Time series measurements from this hypothetical network can be generated by means of a multivariate autoregressive model, as will be shown in a quantitatively precise manner below (Equations 1, 13).

Given only the time series measurements, the problem of interest here is to recover the detailed properties of the network consisting of all the activity properties at each node, and the nature of the direct causal connections, i.e., their strength, direction, and spectral characteristics of the oscillations that are being transmitted.

This study is limited to this type of problem.

Moreover, this rather narrow and simple problem is, to this date, of great interest, as can be seen, for instance, in a recent publication by Plomp et al. (2014), which provides a brief review of methods and offers some benchmark data which will later be used in this present study.

Upon reviewing the history and state of the art in frequency domain causal connectivity studies, there are at least two noteworthy contributions to this field:

1. The noise contribution ratio (NCR) of Akaike (1968). This work has apparently gone unnoticed by most researchers in this field. It has been extensively used and published under other names, in particular by Saito and Harashima (1981), Kaminski and Blinowska (1991), and Baccala et al. (1998). Akaike's NCR method discovers connections (direct and indirect, without distinction), their directionality, and their spectral characteristics.
2. The partial directed coherence (PDC) of Baccala and Sameshima (2001), which is a measure designed to quantify direct connections which are not confounded by indirect paths, their directionality and their spectral characteristics. This is a very widely used measure (cited 672 times at the time of this writing according to “Google-Scholar”), practically considered the “golden standard” when all network properties (and not just part of them) are of interest.

Recently, the PDC has been critically studied by Schelter et al. (2009). They pointed out that the normalization used in the PDC, i.e., the denominator in the PDC formula (see details below), contains influences from the sender node of interest to all receiver nodes, and as a consequence, the PDC decreases in the presence of many nodes, even if the relationship between a sender and receiver of particular interest remains unchanged. The solution to this problem was given in the form of a renormalization of the PDC, using the statistical variance of the strength of the connection.

In this present study, rather the aiming at a re-normalization of the PDC, such as that successfully achieved by Schelter et al. (2009), we reformulate the problem from scratch, estimating the partial coherence under a multivariate autoregressive model, followed by setting all irrelevant associations to zero, other than the particular directional association of interest. This procedure is akin to Pearl's “surgical intervention” for studying causality (Pearl, 2000). This approach gives the isolated effective coherence (iCoh) (Pascual-Marqui et al., 2014).

In the original Baccala and Sameshima paper (Baccala and Sameshima, 2001), a number of simple toy examples were designed to illustrate the superiority of the PDC as compared to other competing methods. Following the same style, we here provide a new simple toy example, which compellingly shows how the PDC can give incorrect information about the strength of a connection, and incorrect information on its spectral characteristics. And we show how the iCoh solves this problem.

To further illustrate the shortcomings of PDC as pointed out by Schelter et al. (2009), both PDC and iCoh are compared below in the analysis of publicly available benchmark data, consisting of somatosensory responses in rats, measured on 15 skull electrodes. The two methods produce dramatically different results, with much reduced PDC values in some cases, consistent with the observation made by Schelter et al. (2009), as discussed above.

In order to test the new iCoh measure as compared to the PDC under more adverse realistic conditions, the following simulation was performed. Five time series with known dynamics were generated, and used as the electrical activity assigned to 5 cortical sites. EEG recordings were computed by solving the forward equations (see e.g., Fuchs et al., 2002; Gomez-Herrero et al., 2008) at 19 scalp electrodes from these sources. The EEG was then given to an inverse solver, namely eLORETA [exact low resolution electromagnetic tomography (Pascual-Marqui, 2007, 2009; Pascual-Marqui et al., 2011)], producing estimated cortical signals which were then used to compute iCoh and PDC. As predicted and as will be shown below, iCoh recovers adequately all the information about the network, whereas PDC also does so, but reporting false results.

In a final example, real human recordings are analyzed, where the iCoh between six cortical regions of interest (ROIs) are calculated and compared under eyes open and closed conditions, using 61-channel EEG recordings from 109 subjects (EEGs from public data base, see Goldberger et al., 2000; Schalk et al., 2004). The ROIs consist of the anterior and posterior cingulate cortices, the inferior parietal lobules, and the dorsolateral pre-frontal cortices. Statistical comparisons for every pair of ROIs, and for every discrete frequency were based on non-parametric randomization of the maximum-statistic (see e.g., Nichols and Holmes, 2002), thus ensuring correction for multiple testing. During eyes closed, the posterior cingulate significantly sends alpha activity to all other regions. During eyes open, the anterior cingulate significantly sends theta-alpha activity to the dorsolateral pre-frontal cortices.

For the sake of reproducible research, the software code implementing the methods discussed here (using lazarus free-pascal “www.lazarus.freepascal.org”), including test data as a simple text file, are freely available at: https://sites.google.com/site/pascualmarqui/home/icoh-isolated-effective-coherence.

## Methods

### The multivariate autoregressive model

As described above, the definition of the iCoh is based on formulating a multivariate autoregressive model, and calculating the corresponding partial coherences after setting all irrelevant connections to zero. All technical details can be found in Pascual-Marqui et al. (2014). For the sake of completeness, a brief presentation is included here. General background and notation on multivariate autoregressive models, frequency domain causality, and spectral density matrices can be found, for instance, in Akaike (1968) and Yamashita et al. (2005).

A stable, stationary multivariate autoregressive model of order *p* ≥ 1, for *q* ≥ 2 time series **X**(*t*) ∈ ℝ^*q*×1^, is written as:

$$X\text{​}\left(t\right)=\sum_{k=1}^{p}A\text{​}\left(k\right)X\text{​}\left(t-k\right)+\;\varepsilon \text{​}\left(t\right)$$

where **A**(*k*) ∈ ℝ^*q*×*q*^ are the autoregressive coefficients, **ε**(*t*) ℝ^*q*×1^ is the innovations (noise) vector, and *t* denotes discrete time.

In general, the autoregressive coefficients [**A**(*k*)]_*ij*_, i.e., the element (*i*, *j*) of the matrices **A**(*k*), quantify the direct causal influence for *j* → *i*. This corresponds to Granger causality (Granger, 1969; Lütkepohl, 2007; Valdes-Sosa et al., 2011).

Given data sampled in discrete time, and given an autoregressive order *p* ≥ 1, the autoregressive coefficients and the innovation covariance matrix can be estimated by any number of methods, one of which is least squares (see e.g., Akaike, 1968). The model order *p* can be estimated by means of Akaike's information criterion AIC (Akaike, 1974).

The frequency domain representation is:

X​(ω)=A​(ω)X(ω)+ε​(ω)

where *X* (ω) ∈ ℂ^*q*×1^, *A* (ω) ∈ ℂ^*q*×*q*^, ∈ (ω) ∈ ℂ^*q*×1^ are the discrete Fourier transforms, and where ω denotes discrete frequency.

From Equation 2, the Hermitian covariance, i.e., the spectral density matrix, is:

$$S_{x}\text{​}\left(\omega \right)={\left(\overset{˘}{A}\text{​}\left(\omega \right)\right)}^{-1}S_{\varepsilon }{\left({\overset{˘}{A}}^{*}\text{​}\left(\omega \right)\right)}^{-1}$$

with :

$$\overset{˘}{A}\text{​}\left(\omega \right)=I-A\text{​}\left(\omega \right)$$

where the superscript “^*^” denotes matrix transpose and complex conjugate, the superscript “−1” denotes matrix inversion, **I** is the identity matrix, and **S**_ε_ ∈ ℝ^*q*×*q*^ is the noise covariance.

### The isolated effective coherence (iCoh)

From the spectral density matrix (Equation 3), the partial coherences (see e.g., Brillinger, 2001) between any pair of nodes (*i*, *j*) can be calculated. The significance of the partial coherence in a very general setting can be found in Radhakrishna Rao (1981). In simple terms, the partial coherence is a measure of association between two complex valued random variables after removing the effect of other measured variables.

The full general equation for the partial coherence as a function of all the autoregressive coefficients contains information on all possible connection paths. Technical details can be found in Equations 8, 9 within Pascual-Marqui et al. (2014). However, in order to “isolate” the direct and directional parts of a connection, all other possible paths must be severed. This is a procedure commonly used in causality analysis, metaphorically known as performing a “surgical intervention” (see e.g., Pearl, 2000).

For this reason, the isolated effective coherence (iCoh) for *j* → *i* is defined under the condition that the only non-zero association between the time series is due to [**Ă**(ω)]_*ij*_ ≠ 0. This requires that all other possible associations be set to zero, i.e.,:

$$\begin{array}{l} {\left[A\text{​}\left(\omega \right)\right]}_{kl}\equiv 0\;,\text{ for all }\left(k,\;l\right)\text{ such that }\\ \;\left(k,\;l\right)\neq \left(i,\;j\right)\text{ and }k\neq l \end{array}$$

and :

$${\left[S_{\varepsilon }\right]}_{kl}\;\equiv 0\;,\text{ for all }\left(k,\;l\right)\text{ such that }k\neq l$$

Note that the diagonal elements of **S**_ε_ and **A** (ω) remain unmodified, since they do not “associate” different nodes.

Emphasis must be placed on the fact that this procedure is meaningful only if the new system with a single association remains stable and stationary.

When the constraints in Equations 5, 6 are applied to the general partial coherence, we obtain the isolated effective coherence (iCoh). In particular, iCoh for *j* → *i* is defined as the squared modulus of the partial coherence between *i* and *j* under the constraints given by Equations 5, 6:

$$\kappa_{i\leftarrow j}\left(\text{ω}\right)=\frac{{\left[S_{\text{ε}}\right]}_{ii}^{-1}{\left|{\left[\overset{˘}{A}\left(\text{ω}\right)\right]}_{ij}\right|}^{2}}{{\left[S_{\text{ε}}\right]}_{ii}^{-1}{\left|{\left[\overset{˘}{A}\text{​}\left(\text{ω}\right)\right]}_{ij}\right|}^{2}+{\left[S_{\text{ε}}\right]}_{jj}^{-1}{\left|{\left[\overset{˘}{A}\left(\text{ω}\right)\right]}_{jj}\right|}^{2}}$$

which clearly satisfies:

$$0\leq \kappa_{i\leftarrow j}\left(\text{ω}\right)\leq 1$$

The detailed, step by step derivations are shown in Pascual-Marqui et al. (2014).

The iCoh can be described as the answer to the following question:

“Given a dynamic linear system characterized by its autoregressive parameters, what would be the equation for the partial coherence if all connections are severed, except for the single one of interest?”

Note that the algorithm for computing the iCoh requires:

1. The estimation of the full, joint, multivariate autoregressive model (Equation 1). This step is performed only once.
2. For any given pair of nodes and any direction such as *j* → *i*, compute Equation 7 using the parameters from step (1).

### The partial directed coherence (PDC) and the generalized partial directed coherence (gPDC)

These definitions are replicated here for the sake of completeness.

The PDC is:

$${\left|{\bar{\text{π}}}_{ij}\left(\text{ω}\right)\right|}^{2}=\frac{{\left|{\left[\overset{˘}{A}\text{​}\left(\text{ω}\right)\right]}_{ij}\right|}^{2}}{{\left[\overset{˘}{A}*\text{ ​}\left(\text{ω}\right)\overset{˘}{A}\text{​}\left(\text{ω}\right)\right]}_{jj}}=\frac{{\left|{\left[\overset{˘}{A}\left(\text{ω}\right)\right]}_{ij}\right|}^{2}}{\sum_{k=1}^{q}{\left|{\left[\overset{˘}{A}\text{​}\left(\text{ω}\right)\right]}_{kj}\right|}^{2}}$$

which corresponds to Baccala and Sameshima (2001), Equation 18 therein.

The gPDC is:

$${\left|{\text{π}}_{ij}^{w}\left(\text{ω}\right)\right|}^{2}=\frac{{\left[S_{\text{ε}}\right]}_{ii}^{-1}{\left|{\left[\overset{˘}{A}\text{​}\left(\text{ω}\right)\right]}_{ij}\right|}^{2}}{\sum_{k=1}^{q}{\left[S_{\text{ε}}\right]}_{kk}^{-1}{\left|{\left[\overset{˘}{A}\text{​}\left(\text{ω}\right)\right]}_{kj}\right|}^{2}}$$

which corresponds to Baccalá et al. (2007), Equation 11 therein.

Note that these measures are not proper partial coherences. The squared modulus of a proper coherence or partial coherence has a value between zero and one, and they do not have a column or row sum value of 1.

### Statistics

In some instances, results will be presented simply as the estimated values of connectivities, without performing an actual statistical test. This type of result is akin to showing the effect size.

In other cases, where specified, statistical tests are carried out based on the method of non-parametric randomization of the maximum-statistic, which has the advantage of correcting for multiple testing, and of not relying on Gaussianity (Blair and Karniski, 1994; Karniski et al., 1994; Nichols and Holmes, 2002; Nichols, 2012).

A brief description of the multivariate non-parametric randomization method follows. Technical details are not included here because they can found in the specialized literature, see e.g., Nichols and Holmes (2002) and the cited literature therein. Consider an example where the data is represented as *X_cki_*, consisting of *i* = 1… *R* variables, measured on *k* = 1… *N* subjects, under two conditions *c* = 1 and *c* = 2. The variables may correspond to cortical spectral power at each voxel and each frequency, or to direct and directed connection strength between each pair of regions of interest and each frequency.

In this example, the aim is the discovery of the variables that are significantly different between the two conditions. For this purpose, the simple variable-by-variable *t*-statistic can be used as a statistical measure of “distance” between the two conditions. Other choices of statistics are equally valid. From the set of “*R*” *t*-statistics (one for each variable), the absolute maximum is chosen. Then its empirical probability distribution is estimated by repeatedly randomizing the conditions “*c*,” and recalculation the maximum-*t*'s under the null hypothesis. This empirical probability gives the threshold with correction for multiple (“*R*” tests) testing, as explained in Nichols and Holmes (2002). The correction is exact (in the sense of Fisher's exact test) for a large number of randomizations, regardless of the original probability distribution of the variables.

### EEG: forward and inverse problems

The equation of electrodynamics that links current density in the brain to scalp electric potential differences is known as the “forward” equation of EEG. This forward problem, which has a well-defined solution, is typically solved with numerical methods.

Simulated EEG is easily created by placing sources of time varying electric neuronal activity at any number of cortical sites, and calculating the electric potential differences on scalp electrodes, by means of the forward equation.

Formally, the forward equation of EEG in discrete form at time instant “*t*” can be written as:

$$\Phi_{t}=KJ_{t}$$

where Φ_*t*_ ∈ ℝ^*N*_*E*_ ×1^ denotes the instantaneous scalp electric potential at *N_E_* electrodes, **J**_*t*_ ∈ ℝ^(3*N*_*V*_)×1^ is the instantaneous current density vector field at *N_V_* cortical voxels (consisting of three components at each voxel), and **K** ∈ ℝ^*N_E_* ×(3*N_V_*)^ is the lead field.

The inverse problem, which consists of estimating the cortical activity (current density vector field) from measured scalp EEG, is known to have no unique solution (see e.g., Helmholtz, 1853; Pascual-Marqui, 2009). This is the reason for the existence of many different inverse solutions found in the literature. In this study, the method known as exact low resolution electromagnetic tomography (eLORETA; Pascual-Marqui, 2007, 2009; Pascual-Marqui et al., 2011) is used for estimating sources in both simulated EEG and for real human EEG measurements. The eLORETA solution has the following generic form:

$${\hat{J}}_{t}=T\Phi_{t}$$

where **T** ∈ ℝ^(3*N_V_*)×*N_E_*^ is the eLORETA pseudoinverse (Pascual-Marqui, 2007; Pascual-Marqui et al., 2011).

In the current implementation of eLORETA, computations are made in a realistic head model (Fuchs et al., 2002), using the MNI152 template (Mazziotta et al., 2001), with the three-dimensional solution space restricted to cortical gray matter, as determined by the probabilistic Talairach atlas (Lancaster et al., 2000). The standard electrode positions on the MNI152 scalp were taken from Jurcak et al. (2007). A total of 6239 cortical gray matter voxels at 5 mm spatial resolution constitute the solution space.

The estimated time varying electric neuronal activity at each cortical voxel (given by **Ĵ**_*t*_ in Equation 12) consists of three time series, one for each moment component of the current density vector (i.e., dipole). In practice, this can be reduced to a single time series, due to the fact that the current density vector is anatomically constrained to have an orientation orthogonal to the cortical surface (see e.g., Baillet et al., 2001). Under this assumption, the 3 × 3 covariance matrix for the current density vector at each voxel must have rank 1, with the dipole orientation given by its largest eigenvector (Mosher et al., 1992; Mosher and Leahy, 1998). This procedure is applied in this study for the estimation of single time series of electric neuronal activity at each voxel. Note that this estimator for the current density vector field orientation is a maximum variance estimator.

## Materials

### A toy example: five time series

Simulated recordings from five time series were generated from the following stable, stationary multivariate autoregressive model of order 2:

$$\begin{array}{l} A\text{​}\left(1\right)=\left(\begin{array}{ccccc} 1.5 & -0.25 & 0 & 0 & 0\\ -0.2 & 1.8 & 0 & 0 & 0\\ 0 & 0.9 & 1.65 & 0 & 0\\ 0 & 0.9 & 0 & 1.65 & 0\\ 0 & 0.9 & 0 & 0 & 1.65 \end{array}\right);\\ A\text{​}\left(2\right)=\left(\begin{array}{ccccc} -0.95 & 0 & 0 & 0 & 0\\ 0 & -0.96 & 0 & 0 & 0\\ 0 & -0.8 & -0.95 & 0 & 0\\ 0 & -0.8 & 0 & -0.95 & 0\\ 0 & -0.8 & 0 & 0 & -0.95 \end{array}\right);\\ S_{\text{ε}}=I \end{array}$$

The direct causal directed connections between nodes are illustrated as arrows in Figure 1.

Assuming a Gaussian distribution for the noise (zero mean, unit variance, as shown in Equation 13), 25600 time samples were generated (after discarding the first 1000 time samples) and used for all estimation procedures.

Assuming that the times series are sampled at 256 Hz, the main spectral properties of this network, by construction, are the following:

1. Node 1 in isolation oscillates at peak frequency 28 Hz.
2. Node 2 in isolation oscillates at peak frequency 16 Hz.
3. Nodes 3, 4, and 5 in isolation oscillate at peak frequency 28 Hz.
4. Nodes 3, 4, and 5 are receiving identical information from node 2.

### Simulated EEG

In a different setting, the five time series generated in the previous subsection were used as the time varying electric neuronal activities at the following cortical locations:

1. Superior frontal gyrus (left), BA 10: *X* = −25, *Y* = 65, *Z* = −5
2. Middle Occipital Gyrus (right), BA 18: *X* = 20, *Y* = −100, *Z* = 5
3. Post-central Gyrus (left), BA 3: *X* = −50, *Y* = −20, *Z* = 60
4. Middle Temporal Gyrus (left), BA 21: *X* = −65, *Y* = −15, *Z* = −15
5. Middle Temporal Gyrus (right), BA 21: *X* = 70, *Y* = −20, *Z* = −10

where (X, Y, Z) denotes the MNI coordinates in millimeters, and BA denotes Brodmann area.

**Figure 2:**
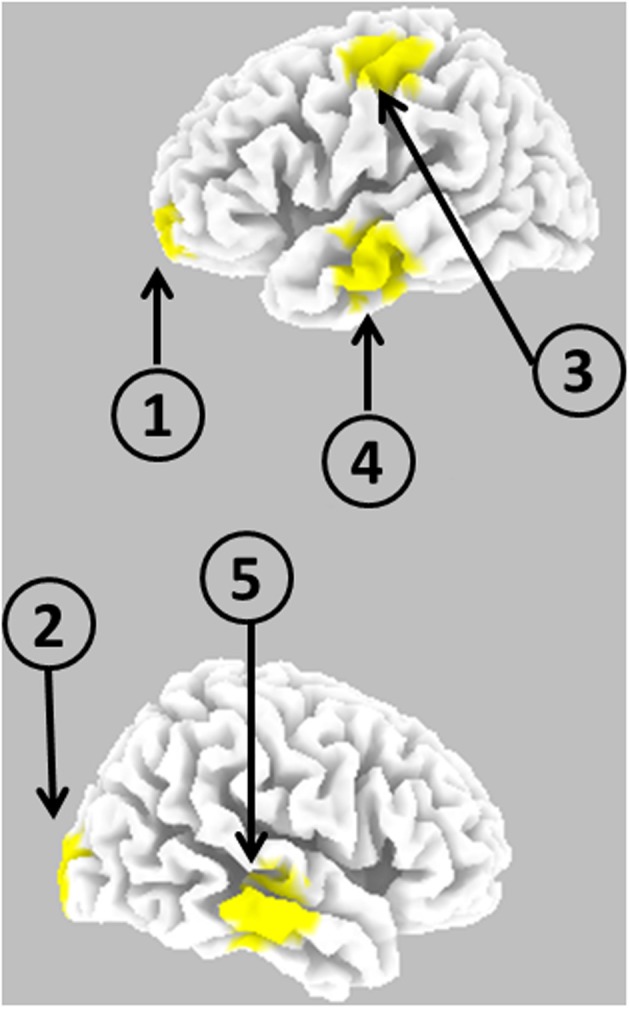
**Schematic representation of the anatomical locations of five cortical point sources used for generating EEG**.

Figure 2 illustrates the five cortical locations.

Use was made of the forward equations previously explained for generating EEG recordings at 19 scalp electrodes, corresponding to the 10/20 electrode placement system. In this generation process, two relatively large sources of noise were added:

1. Biological noise, where independent and identically distributed uniform [0, 0.2] random values were assigned to the current density at each cortical voxel and at each time sample.
2. Measurement noise, where independent and identically distributed uniform [0, 0.2] random values were multiplied by the potential at each electrode and time sample, and added to potential.

### Rat EEG

EEG recorded at 2 kHz sampling rate from 15 electrodes placed directly onto the skull of rats, during a somatosensory experiment, are publicly available from Plomp et al. (2014). A single recording from their repository, the data from the file named “RN060915A2_STIMD,” was taken for analysis. This corresponds to the average evoked response for one particular animal, with unilateral whisker stimulation. The recording starts at −60 ms relative to stimulus onset, and has a total duration of 180 ms.

### Human EEG recordings

Real human EEG recordings under eyes open and closed conditions, using 64-channel EEG recordings from 109 subjects, are publicly available from Goldberger et al. (2000), Schalk et al. (2004). Each recording (218 in total) consists of 1 min. EEG, sampled at 160 Hz. Three electrodes (T9, T10, and Iz) were discarded for analysis, because they were spatial outliers relative to the other 61 electrodes that cover the scalp in an approximate uniformly distributed manner.

## Results

### A toy example: five time series

**Figure 3:**
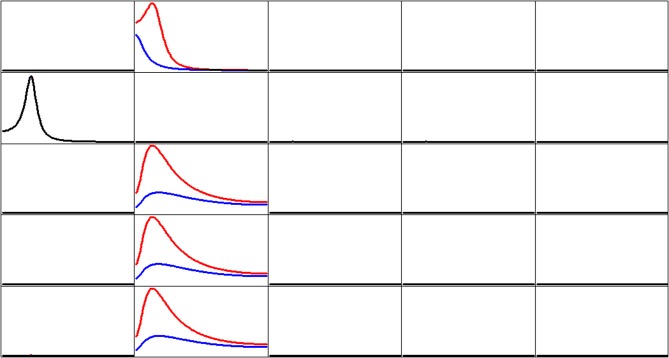
**Estimated connectivity properties for the network in Figure 1.** Isolated effective coherence (iCoh) shown in RED, and the generalized partial directed coherence (gPDC) shown in BLUE. Overlap of both curves is shown in BLACK. Vertical axis: 0 to 1. Frequency axis: 1 to 127 Hz. Columns are senders, rows are receivers. Coherence peak in column 1 occurs at 28 Hz. Coherence peak for iCoh in column 2 occurs at 16 Hz. Coherence peak for gPDC in column 2, row 1 occurs at 1 Hz; and Coherence peak for gPDC in column 2, rows 3, 4, and 5 occur at 23 Hz.

Figure 3 shows the iCoh (Equation 7) and the gPDC (Equation 10) calculated for the network in Figure 1. In both cases, the same estimated multivariate autoregressive model of order *p* = 3 was used. The results were essentially identical for autoregressive order *p* = 2.

Of importance to note in Figure 3: the two methods give very different results with respect to node #2 as sender (column 2).

### Simulated EEG

**Figure 4:**
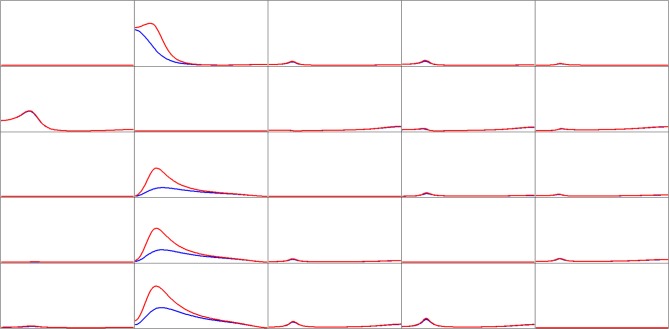
**Estimated connectivity properties for simulated EEG signals (see Figure 2).** Isolated effective coherence (iCoh) shown in RED, and the generalized partial directed coherence (gPDC) shown in BLUE. Vertical axis: 0 to 1. Frequency axis: 1 to 127 Hz. Columns are senders, rows are receivers. Coherence peak in column 1 occurs at 28 Hz. Coherence peak for iCoh in column 2 occurs at 16 Hz. Coherence peak for gPDC in column 2, row 1 occurs at 1 Hz; and Coherence peak for gPDC in column 2, rows 3, 4, and 5 occur at 23 Hz.

The simulated EEG time series for 19 scalp electrodes, using as generators the five cortical locations described in the materials section (Figure 2), with time dynamics from the previous example, were analyzed with eLORETA. We emphasize that this EEG was corrupted with relatively large amounts of additive biological and measurement noise. eLORETA was computed at all 6239 cortical voxels. However, connectivity computations are presented for the estimated electrical activities at the same cortical sites as in Figure 2. Figure 4 shows the estimated iCoh and gPDC. In both cases, the same estimated multivariate autoregressive model of order *p* = 3 was used. The results were essentially identical for autoregressive order *p* = 2.

Of importance to note in Figure 4:

1. Ideally, Figure 4 should be identical to Figure 3. This is the case to a very good approximation from a qualitative point of view, despite the use of estimated signals using eLORETA, from as few as 19 electrodes, and corrupted with relative high levels of biological and measurement noise.
2. The two methods give very different results with respect to node #2 as sender (column 2).

### Rat EEG

The average somatosensory evoked response for one rat was analyzed with a multivariate autoregressive model of order *p* = 8, based on the model order determined in the original publication. Although this data is clearly not stationary, it was analyzed as such in the original publication (Plomp et al., 2014), using a recursive least squares (RLS) algorithm with a forgetting factor, in order to implement a time varying version of the autoregressive model. The only particular and differentiating feature in this current study is that there is no forgetting factor.

**Figure 5:**
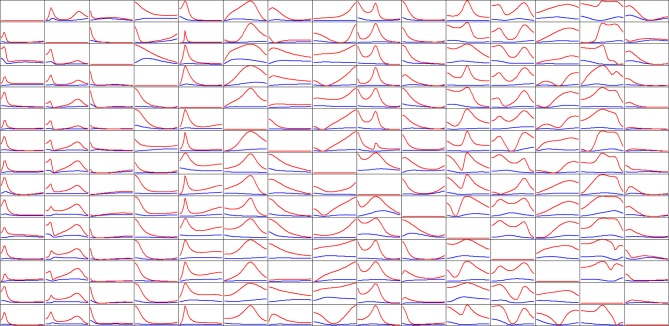
**Estimated connectivity properties for rat EEG recordings from 15 skull electrodes.** Isolated effective coherence (iCoh) shown in RED, and the generalized partial directed coherence (gPDC) shown in BLUE. Vertical axis: 0 to 1. Frequency axis: 7.8 to 250 Hz. Columns are senders, rows are receivers.

But regardless of these considerations, the sole purpose of this rat data analysis is to show the extreme differences in network properties estimated by iCoh and gPDC, as shown in Figure 5.

Of importance to note in Figure 5: the extremely low connectivity values produced by gPDC as compared to iCoh, and the number of missing spectral peaks in gPDC as compared to iCoh.

### Human EEG recordings

EEGs recorded from 109 subjects under eyes open and eyes closed conditions were analyzed. Resting state, awake, eyes closed EEG is characterized by the presence of alpha rhythm, as compared to the eyes open condition.

**Figure 6:**
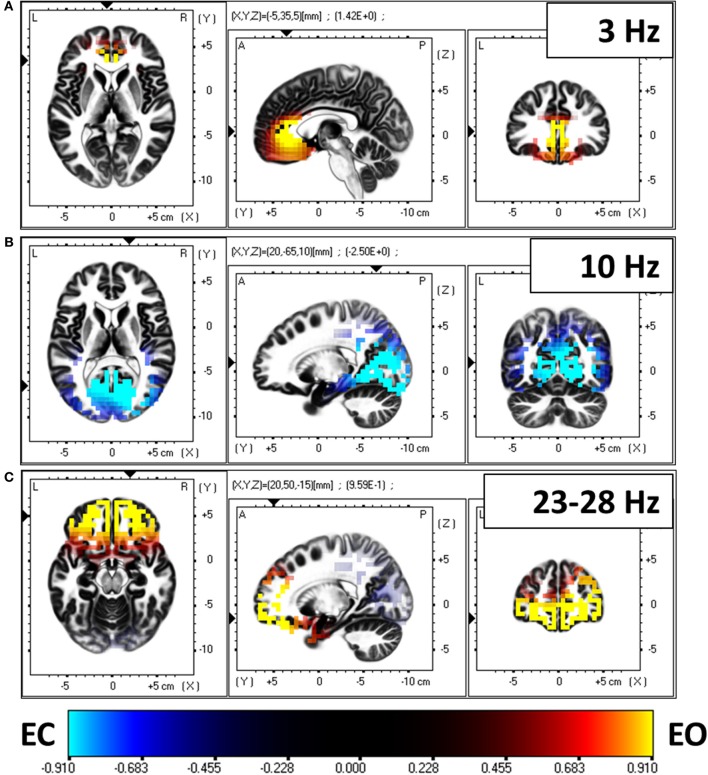
**Comparison of electric neuronal activity (eLORETA) between eyes open and closed conditions.** A Log-F-ratio statistic with correction for multiple testing was used, with corrected *p* = 0.05 at LogF = 0.91. Eyes open is characterized by significantly stronger activity in frontal cortical regions oscillating at 3 Hz **(A)** and in the beta band 23–28 Hz **(C)**. Eyes closed is characterized by significantly stronger activity in occipital cortical regions oscillating at 10 Hz **(B)**.

In a first analysis step, the spectral density of electric neuronal activity throughout the cortex was calculated with eLORETA at all 6239 cortical voxels. The technical details on calculating cortical activity spectra can be found in Frei et al. (2001). A voxel by voxel, frequency by frequency comparison between eyes open and closed conditions was performed. Figure 6 shows the three main statistically significant results. Eyes open is characterized by significantly stronger activity in frontal cortical regions oscillating at 3 Hz and in the beta band 23–28 Hz. Eyes closed is characterized by significantly stronger activity in occipital cortical regions oscillating at 10 Hz.

In a second analysis step, time series of electric neuronal activity were estimated with eLORETA at 6239 cortical voxels, from which six cortical regions of interest were used for the connectivity analyses. This procedure was applied to the EEGs recorded in 109 subjects, under eyes open and eyes closed conditions. The regions of interest are:

1. Anterior Cingulate, BA 32: *X* = 0, *Y* = 45, *Z* = 10
2. Posterior Cingulate, Precuneus, BAs 23, 31: *X* = 0, *Y* = −50, *Z* = 30
3. Inferior Parietal Lobule (left), BA 40: *X* = −45, *Y* = −45, *Z* = 50
4. Inferior Parietal Lobule (right), BA 40: *X* = 45, *Y* = −45, *Z* = 50
5. Dorsolateral Pre-frontal (left), BA 10: *X* = −40, *Y* = 40, *Z* = 25
6. Dorsolateral Pre-frontal (right), BA 10: *X* = 40, *Y* = 40, *Z* = 25

**Figure 7:**
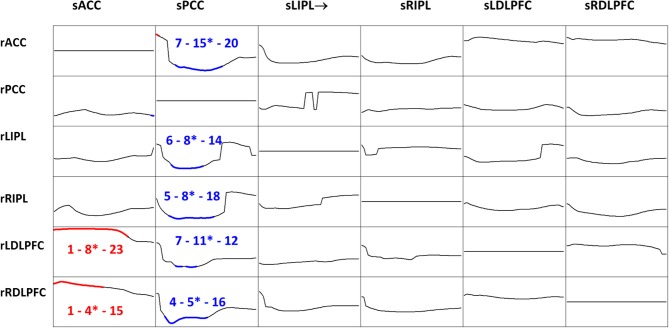
**t-Statistics comparing eyes open minus eyes closed iCoh for 109 subjects, in six regions of interest: anterior cingulate, posterior cingulate, left and right inferior parietal, and left and right dorsolateral pre-frontal cortices.** Frequency axis: 1 to 30 Hz. Corrected *p* = 0.05 was at t-threshold = 4.3, with vertical axis: −7 to +7. Blue color denotes eyes closed significantly larger, red color denotes eyes open significantly larger. The three numbers indicate the frequencies (Hz) for the significant results: start, end, and the most significant oscillation indicated with a superscript “^*^.” Columns are senders (prefix “s”), rows are receivers (prefix “r”).

iCoh was estimated for the six time series, in the 218 recordings, using an autoregressive order *p* = 7, which corresponds to the median order for all EEG recordings based on Akaike's AIC (see subsection “The multivariate autoregressive model”). A statistical comparison between eyes open and eyes closed conditions was carried out, for each frequency, for each pair of regions of interest, and for each direction of connection. The significant differences at probability 0.05 with correction for multiple testing, are shown in Figure 7.

**Figure 8:**
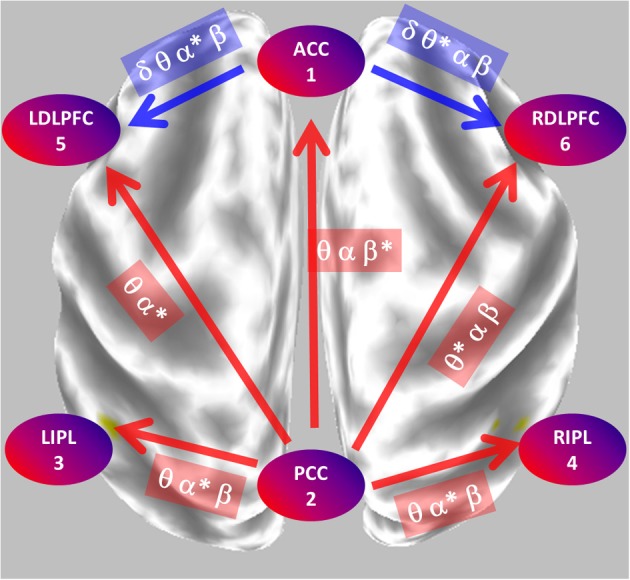
**Summary of the main statistically significant results comparing the network properties between eyes open and closed conditions.** During eyes closed, the posterior cingulate significantly sends mostly alpha oscillations to all other regions. During eyes open, the anterior cingulate significantly sends mostly theta-alpha oscillations to the dorsolateral pre-frontal cortices. PCC, posterior cingulate cortex; ACC, anterior cingulate cortex; LIPL, RIPL, left and right inferior parietal lobule; LDLPFC, RDLPFC, left and right dorsolateral pre-frontal cortex.

Figure 8 summarizes the main statistically significant results. During eyes closed, the posterior cingulate significantly sends activity to all other regions. During eyes open, the anterior cingulate significantly sends activity to the dorsolateral pre-frontal cortices.

## Discussion

The results corresponding to the toy example with five time series demonstrate very clearly a major problem with the partial directed coherence (PDC; Baccala and Sameshima, 2001) as well as with its generalized version (gPDC; Baccalá et al., 2007). By construction, node 2 sends identical information to nodes 3, 4, and 5. And yet the gPDC gives very different results. Moreover, the frequency responses of the gPDC are incorrect, with one missing spectral peak and other peaks at incorrect frequencies.

This type of problem was already pointed out by Schelter et al. (2009), and here we show a compelling example of how severe it can be.

In contrast, the isolated effective coherence (iCoh) introduced in this study recovers and reports correctly all the network properties for the toy example.

The results corresponding to the evoked response recordings from an animal experiment (Plomp et al., 2014) demonstrate that gPDC and iCoh can give very different results with real experimental data. Because of the complex biological nature of the data, the ground truth is not unambiguously known. Regardless, this demonstrates that when there are many nodes (15 in this case), the gPDC can give very low connectivity values and almost featureless spectral properties as compared to iCoh.

By its very nature and definition, the gPDC does not report information on a particular direct directed connection. Instead, it is affected by many other connections, in such a way that it can report incorrect values and spectral properties of the “sender-receiver” pair of interest. The iCoh solves this problem, by its very nature and definition (Pascual-Marqui et al., 2014).

In the neurosciences, one of the most interesting applications of a method such as the iCoh is the elucidation of effective cortical connections based on measurements of electric neuronal activity. However, these are extremely invasive measurements. In order to solve this problem non-invasively, one possible approach is to use scalp EEG measurements, and to estimate with an inverse solution the electric neuronal activity at any number of cortical locations. It is then very important to prove that the estimated time series are of sufficient quality to calculate iCoh reliably.

This was the aim of the experiment with simulated EEG. Cortical signals were used for computing EEG, which was then analyzed with the eLORETA inverse solution (which has no prior information about the locations or about the dynamics of the actual sources). Despite the low spatial resolution of eLORETA, and despite the use of only 19 scalp electrodes, iCoh was estimated very reliably. This is partly due to the fact that a measure such as iCoh separates rather well instantaneous and lagged connections, especially if the instantaneous connections are mediated by the noise covariances, which explicitly do not affect iCoh (see Equations 6, 7). However, the low spatial resolution of eLORETA will mix the autoregressive coefficients, as is shown in Gomez-Herrero et al. (2008). Both Gomez-Herrero et al. (2008) and Faes et al. (2013) propose solutions to this problem, which can be applied also to eLORETA signals.

Finally, an eLORETA-iCoh study was performed on real human EEG which is available from a public repository (Goldberger et al., 2000; Schalk et al., 2004). The research aim here was to search for differences in brain function between two resting states, namely eyes open and eyes closed. Two aspects of brain function were explored:

1. The cortical location of the generators of different oscillatory activity (functional localization).
2. The network properties among a group of six very important cortical sites (functional “effective” connectivity).

This type of study is of interest in understanding the resting state of the brain. In particular, in understanding the functional role of the alpha rhythm (Knyazev et al., 2011; Klimesch, 2012; Sigala et al., 2014), and in understanding the functional changes during the eyes open condition (Jao et al., 2013). The results show that eyes closed alpha activity was localized to occipital areas, while delta and beta activities were located in frontal cortical regions. With respect to the network properties, the iCoh analysis demonstrated that the posterior cingulate cortex is a major sender of mainly alpha oscillations to all other regions. Interestingly, during eyes open, this function is turned off, and the anterior cingulate activates as a sender of mainly theta-alpha oscillations to the dorsolateral pre-frontal cortices.

## Outlook and limitations

The iCoh method can be extended to other conditions, different from the particular ones considered here. For instance, instantaneous connections such as those considered in the generalized autoregressive model of Faes et al. (2013) can be directly applied to iCoh. Moreover, since iCoh solely depends on the estimated autoregressive coefficients and the noise variances, these parameters can be estimated under non-stationary, time-varying conditions, as for example in Plomp et al. (2014).

However, if the actual dynamics are non-linear or simply do not follow a linear autoregressive model, then iCoh might be invalid. Non-linear causality measures have been reviewed in Marinazzo et al. (2011), where a novel method is proposed: “kernel Granger causality.” Another method is the phase slope index (Nolte et al., 2008), which is of a more non-parametric nature, not relying on the parametric form of the linear autoregression. However, these two alternative methods do not distinguish the direct or indirect nature of the connections.

Interestingly, a very recent book entitled “Directed Information Measures in Neuroscience” (Wibral et al., 2014) barely deals with methods that reveal all properties of a neural network, namely the spectral content of information flow, the direct or indirect nature of the connections, and the actual direction. One exception is a single chapter that refers to the PDC of Baccala and Sameshima (2001), and to Geweke's method (Geweke, 1984) (which is based on the predictive approach of Granger).

In our present study, the method of Geweke was not studied, and certainly deserves more attention in future research. However, we note that Geweke's method has been criticized elsewhere (Chen et al., 2006) because it often produces negative connectivity values that render it meaningless.

It is important to emphasize that the EEG simulation example presented here is very limited, and only constitutes a “proof of principle,” since the cortical signals used for analysis were close to the actual locations of the sources. The effect of the choice of the number of regions of interest and of their locations relative to the actual unknown active network requires further study.

One common problem in all models that depend on fitting a multivariate autoregressive model is the curse of dimensionality: for a large number of nodes and for a high autoregressive order, the number of parameters to be estimated can be too large to produce reliable estimators. One possible solution is the estimation of sparse multivariate autoregressions as developed by Valdes-Sosa et al. (2005). Alternatively, stable high dimensional autoregressive models can be successfully estimated under spatio-temporal constraints, such as those considered by Jiménez et al. (1995).

The eLORETA method was used in this study. Other inverse solutions can be used. The only requirement is that the selected method needs to be capable of correct estimation of the neuronal current density. This was the reason for choosing eLORETA, because it is an improvement over the previous related tomographies known as LORETA (Pascual-Marqui et al., 1994) and sLORETA (Pascual-Marqui, 2002), which have received considerable and substantial validation (Pascual-Marqui et al., 2011). We note that all these techniques can equally be applied to MEG measurements as well.

There is a severe limitation in the use and interpretation of all connectivity measures (including iCoh) if they are applied to scalp EEG signals. In this case, the results should never be interpreted as representing cortical connections. The reason is that cortical activity does not project radially onto the scalp (see e.g., Lehmann et al., 2006, 2012). This problem applies to all EEG analyses that naively map scalp measurements and features onto the underlying cortex, which in general produce incorrect results.

In conclusion, iCoh is most certainly not intended as the general solution to the problem of identifying network properties. It is a very simple and particular measure for correctly assessing direct connections that causally transmit oscillatory information between nodes, under the assumption of a linear autoregressive model. It is distinct from the PDC method of Baccala and Sameshima (2001); Baccalá et al. (2007), which can produce incorrect results.

### Conflict of interest statement

The authors declare that the research was conducted in the absence of any commercial or financial relationships that could be construed as a potential conflict of interest.
